# Quantitative fatty acid signature analysis (QFASA) to explore the diet of the plateau pika (*Ochotona curzoniae*) during winter on the Qinghai–Xizang (Tibetan) plateau

**DOI:** 10.1007/s00442-026-05893-7

**Published:** 2026-04-09

**Authors:** Min Li, Zengguang Jin, Lukasz Oldakowski, Shien Ren, Baoguo Li, Li Li,, Jazmin Osorio-Mendoza, Yang Jiao, Siqi Yong, Dehua Wang, Yanming Zhang, Guanghou Shui, John R. Speakman

**Affiliations:** 1 Shenzhen Key Laboratory of Metabolic Health, Center for Energy Metabolism and Reproduction, Shenzhen Institute of Advanced Technology, Chinese Academy of Sciences, Shenzhen, 518055 People’s Republic of China; 2 State Key Laboratory of Molecular Developmental Biology, Institute of Genetics and Developmental Biology, Chinese Academy of Sciences, Beijing, 100101 People’s Republic of China; 3 University of Chinese Academy of Sciences, Beijing, 101408 People’s Republic of China; 4 Northwest Institute of Plateau Biology, Chinese Academy of Sciences, Xining, 810008 People’s Republic of China; 5 Tianjian Laboratory of Advanced Biomedical Sciences, Zhengzhou University, Zhengzhou, People’s Republic of China; 6 Institute of Biological and Environmental Sciences, University of Aberdeen, Aberdeen, AB24 2TZ Scotland, UK; 7 School of Life Science, Shandong University, Qingdao, 266237 People’s Republic of China; 8 Shenzhen University of Advanced Technology, Shenzhen, Guangdong People’s Republic of China

**Keywords:** Coprophagy, Elevation gradient, Winter survival, Niche partitioning, Fatty acid biomarkers, QFASA

## Abstract

The plateau pika, a keystone species of the Tibetan Plateau, is also widely poisoned as an agricultural pest. An intriguing observation is that pika are more abundant where their main competitor (domestic yak) is also most abundant. A recent study showed that this may be because pika supplement their diet in winter by consuming yak feces. Here we used quantitative fatty acid signatures analysis to explore pika diet and detect the possibility of coprophagy. We collected fat tissue from pika at 3 sites of varying elevation, and compared the abundance of 10 fatty acids (FA) in their body fat with those in their main potential foods (grass, roots and feces). We used an optimization program to establish the best-fit diet to the observed FA distributions, using mouse fat as a negative control. Pika fat FA signatures matched plateau foods far better than mouse fat. Faeces consumption doubled as the elevation of the site increased from 12.8% at the lowest site to 26.8% at the highest. In contrast consumption of roots showed the opposite pattern, comprising 32% at the lowest elevation and falling to virtually zero at the highest. That might reflect the time that the surface ground is frozen inhibiting the ability to dig for roots at higher elevation sites. Our data support the previous suggestion that pika eat yak feces, potentially giving them an over-winter survival advantage increasing their populations where yak are abundant. The full extent of coprophagy however requires more data in a larger population sample.

**Supplementary Information:**

The online version contains supplementary material available at 10.1007/s00442-026-05893-7.

## Introduction

Understanding trophic relations is important for grasping the multifaceted ecologies of important communities, including those on the Qinghai–Tibetan plateau (QTP) in western China. The QTP spans roughly 2.5 M square kilometers with an average elevation exceeding 4000 m, serving as the source of major Asian rivers, such as Yellow, Yangtze, and Mekong. It also acts as a major global reservoir of frozen fresh water. Dominated by high elevation montane grasslands, which cover about 70% of the land surface, the QTP faces rapid climate change, with ambient temperatures rising about 4 × faster than elsewhere in China (Liu and Chen 2000). This accelerated warming is causing glaciers to retreat and permafrost to melt (Liu and Chen 2000; Ni 2000; Cheng and Wu 2007), raising concerns about the ecosystem integrity, stability and future of the water reserve, and impacts on human infrastructure (Hjort et al. 2022; Ran et al. 2022). Here we elucidate some aspects of the complex trophic interactions between two herbivore competitors that dominate the high alpine meadow ecosystem on the plateau: the yak (*Bos grunniens*) and the plateau pika (*Ochotona curzoniae*). The meadows of the plateau are extensively grazed by domestic yak tended by semi-nomadic Tibetan herders. Alongside these domestic animals the region hosts a variety of endemic species, including the plateau pika, a small and abundant lagomorph that is widely distributed across the plateau. The plateau pika is regarded as a keystone species for the plateau ecosystem (Smith and Foggin 1999) because its burrows provide homes to a variety of small birds (Lai and Smith 2003) and lizards (Zhao et al. 2020), it creates microhabitats that encourage plant diversity and it serves as prey for many plateau predators (Smith and Foggin 1999). Moreover, its burrows facilitate water infiltration reducing the risk of watershed level impacts on plateau hydrology (Wilson and Smith 2015). At low burrow densities the pika burrowing may provide hydrological advantages (Guo et al. 2012) and increase organic matter (Li and Zhang 2006). However, when pika populations reach high densities (up to about 350 individuals per hectare) they can degrade the grasslands (referred to as "black soil": (Li and Huang 1995; Dong et al. 2013)) and reduce forage availability for domesticated animals (Liu et al. 1980; Fan et al. 1999; Han et al. 2008). Concerns over the negative impacts of pika led to systematic poisoning campaigns initiated in the late 1950s (Liu et al. 1980; Fan et al. 1999), though the effectiveness of these campaigns has been questioned (Pech et al. 2007). Despite the poisoning, by 2005, it was estimated that up to 30% of the plateau meadows were degraded (Chen 2005) and five years later this had not improved (Harris 2010).

Interestingly, despite being considered competitors, pika and yak populations are positively correlated, and survival is higher where vegetation cover is reduced (Wangdwei et al. 2013). Experimental manipulations of grazing densities have established that pika populations become higher in grazed than ungrazed plots (Badingquiying et al. 2018). The reasons for this positive association are not entirely clear, but one hypothesis is that in grazed areas, where the grass is shorter, pika can more easily detect and hence evade predators (Arthur et al. 2008), such as Tibetan wolves (*Canis himalayensis*), foxes (*Vulpes ferrilata*) (Harris et al. 2014), and polecats (*Mustela eversmanii*). Recently, Speakman and colleagues discovered that pika might also benefit from the presence of yak in an unexpected way: they may eat yak feces as an additional abundant food resource during the winter, when the grass is dry and parched (Speakman et al. 2021). Evidence for this interspecific coprophagy came from several sources. First, there was a strong convergence of pika and yak microbiota during winter, that was absent in the summer. They recovered yak DNA from 22.7% of pika stomach contents, and finally they captured several videos of pika eating yak feces. However, it remains unclear whether this behavior is widespread among pika, or if it is an occasional survival strategy with limited ecological implications.

Quantitative fatty acid signature analysis (QFASA) is a method for reconstructing animal diets from the fatty acid composition of their stored triglycerides (Galloway and Budge 2020). Food sources vary in their fatty acid signatures, and although there are well-established enzymatic pathways that allow animals to convert between different fatty acids, this process is energetically costly. Therefore, animals tend to store fat with minimal conversion, meaning the fatty acid profile in their body fat can reflect their diet over time. A benefit of this approach is that it is relatively insensitive to occasional individual dietary acts, but rather provides a longer-term average view of the diet and hence the importance of specific dietary components. QFASA has been validated and widely used to assess the diets of animals, especially where other techniques are impractical—including cetaceans like humpback dolphins (*Sousa chinensis*) (Xie et al. 2022), minke whales (Olsen and Grahl-Nielsen 2003) and beluga whales (*Delphinapterus leucas*) (Dahl et al. 2000; Choy et al. 2020), seals (Iverson et al. 1997; Walton et al. 2000; Budge et al. 2004; Nordstrom et al. 2008; Knox et al. 2019) and polar bears (*Ursus maritimus*) (Grahl-Nielsen et al. 2003; Thiemann et al. 2008). The major application of this technique has been in marine ecosystems, although there are some recent terrestrial examples, including Collembola in soils (Kühn et al. 2020). Here, we employed QFASA to quantify the contribution of yak faeces to the winter diet of plateau pikas at three sites across an elevation gradient on the QTP. Our results highlight the potential of QFASA for reconstructing the diets of terrestrial species, thereby validating its application beyond traditional marine contexts.

## Materials and methods

### Description of the field sites

Three sampling sites in this study exhibit a typical plateau continental climate with long, cold winters. Site F (low elevation): 3,200 m ASL, Menyuan county: 37.59 N, 101.33 E, has a cold-temperate humid winter climate, with mean winter temperatures ranging from − 10 °C to − 2 °C, frequent snowfall, moderate precipitation, and strong winds.; Site H (mid elevation): 3,500 m ASL ∼3.6 km north of Qinghai lake, Gangcha county: 37.25 N, 100.29 E, experiences severe cold and dry winters, with mean winter temperatures around − 17.5 °C, very low precipitation, and persistent strong winds.; Site X (high elevation): 4,000 m ASL, Guolou district Maqin county: 34.46 N, 100.36 E, shows an extremely cold and long winter with high snowfall, strong wind, hypoxia, and mean winter temperatures below − 15 °C, consistent with the harsh alpine environment at high elevation(Liu et al. 2017; Wan et al. 2017).

### Animals and adipose tissue sampling

20 Plateau pikas (Site F: n = 8; Site H: n = 7; Site X: n = 5) from 3 different sites were live-trapped using nooses at the burrow entrances during the winters of 2017–2018. Following capture, the animals were humanely euthanized by carbon dioxide inhalation. All samples were immediately transported to the laboratory on dry ice and stored at − 80 °C prior to processing. Adipose tissue was dissected from the euthanized pikas, and the tissue samples were subsequently stored at − 80 °C for long-term preservation. All experimental procedures were reviewed and approved by the Ethical Review Board of the Institute of Zoology, Chinese Academy of Sciences, Beijing (Approval Number: IOZ20150065-1).

### Field sampling

A quadrat method was used for the collecting of samples from the field during December, 2021. The length and width of the quadrat were both 0.5 m. The quadrat was randomly positioned by throwing a tennis ball at different sampling sites, then grass and root samples were collected in the quadrat around where the ball landed. We did not separate the collected vegetation into different plant species but rather just collected a pooled sample of all the vegetation in the quadrat. Dry yak feces were collected around the quadrat. We collected 8 independent samples of each of these potential food sources at 3 sites.

We collected eight independent samples of each of these potential food sources at three sites of varying elevation.

### Mouse samples

C57BL/6N mice were fed with a low-fat diet (10% fat, 10% protein, D14071613, Research Diets, New Brunswick, USA), and dissected at 33 weeks of age. Subcutaneous white adipose tissue (subWAT) was collected, and the fatty acid composition was measured. This acted as a negative control (see below).

### Analysis of FAs

Fatty acids in adipose tissue, grass, root and yak feces were extracted using the Folch method (Folch et al. 1957). Briefly, lipids were extracted from 10–20 mg adipose tissue samples, or from about 100 mg grass, root and yak feces samples using a chloroform–methanol (2:1, vol/vol) procedure. Our approach assumes that intra-individual/sample variation is negligible. Each weighed sample was spiked with 10 μL of the internal standard (ISTD) Nonadecanoic acid (1 mg/mL). The extracted lipids were saponified with 500 μL of 0.5 N NaOH at 80 °C for 2 h. Then, 500 μL of 14% BF3 in methanol was added for methylation at the same temperature for 30 min. After cooling, 200 μL of 0.9% NaCl water solution and 500 μL of *n*-hexane were added. The samples were then centrifuged at 12,000 rpm for 5 min at 4 °C. The upper organic phase was collected and dried over anhydrous sodium sulfate. The extract was diluted with n-hexane (1:10) and subjected to gas chromatography analysis, using an Agilent 7890B GC equipped with an Agilent 7693A autosampler and an Agilent 7000D mass spectrometry detector. FAMEs were separated using a J&W DB-Fast FAME capillary column (30 m × 0.25 mm × 0.25 μm film thickness) (Agilent Technologies; Santa Clara, CA, USA). The split ratio was 10:1, and the helium flow rate and temperature ramp were as described by Opazo-Ríos et al. (2022). The initial temperature was set at 50 °C, with a final temperature of 240 °C, and the injector temperature was 240 °C. Fatty acids were identified by comparing the retention times of FAMEs with those of a standard FAME mixture (37 Component FAME Mix, ANPEL, CDAA-252795-MIX-1 ml), and fatty acid concentrations were calculated based on their peak areas.

### Correspondence of the food signatures to the body fat signatures – optimisation program

We wrote a simple optimisation program in Python to predict the diet composition from the stored body fat (Python code available in supplementary materials). This program is based on three major assumptions: first, it is assumed that when the foods are digested there is no discrimination in the fatty acids between those absorbed and those excreted. Second, it is assumed fatty acids are not inter-converted after consumption. Third, it is assumed that when the fatty acids have been absorbed there is also no discrimination between those directed toward storage and those directed to be metabolized. These assumptions are all potentially violated and need to be considered. We discuss below approaches to account for their violation. Given these assumptions the program cycled through all the possible food combinations and calculated the diet that generated the lowest absolute deviation to the observed stored fat. To illustrate how this works, consider an animal eating two food types, the fat composition of which includes only 2 fatty acids. Food 1 contains 100% of fatty acid 1 and 0% of fatty acid 2, while food 2 has the reverse configuration containing 0% of fatty acid 1 and 100% of fatty acid 2. If the body fat stores of the animal contain 38% of fatty acid 1 and 62% of fatty acid 2, then the dietary combination that minimizes the deviation to the stored fat is to get 38% of its fatty acids from food 1 and 62% of food 2. In this simple case it is possible to get an exact fit. However, consider now a situation where food 1 contains 80% of fatty acid 1 and 20% of fatty acid 2, while food 2 contains 60% of fatty acid 1 and 40% of fatty acid 2. If the body fat stores still contain 38% of fatty acid 1 and 62% of fatty acid 2, then it is not possible to get an exact fit because in both foods fatty acid 1 is more abundant than fatty acid 2, yet in the body fat the reverse is the case. In this situation the least deviant diet is consumption of only diet 2. In our actual situation there are three food types and 10 fatty acids. So the fitting process is more complex but the principle is the same. The program cycled through all the potential combinations of the three foods to find the combination of foods (under the above assumptions) with the minimum absolute deviation to the fatty acid distribution observed in the fat stores. In theory there might be multiple solutions that come close to the same minimum. To evaluate if that is the case, we plotted the relationships between the deviations and the % contributions of the iterated diets. In none of the cases were multiple solutions indicated. Given animals tend to store a subset of the available fatty acids then it is possible by fitting this model to generate a diet prediction for any animal, whether they were feeding on the foods or not. We therefore used the fatty acid distributions in the fat of five laboratory mice feeding on a completely different diet as a negative control. We compared the summed deviation between the pika fat and the food sources and the mouse fat and the food sources. We reasoned that since we knew these mice were not feeding on any of these foods then the summed deviations for the mice should be much larger than the summed deviations for the pika. If they were not then we could not place much faith in the diet predictions for the pika.

### Deriving calibration coefficients and refined analysis

The above model is simplistic because of the three assumptions regarding the absence of discrimination at the points of excretion/absorption and storage/metabolism, and the absence of inter-conversion. To account for this, it has been suggested that to use the fatty acid signature approach one needs to perform feeding studies that quantify these discrimination effects. Derived ‘calibration coefficients’ from these experiments can then be applied to obtain more realistic dietary composition information. Bromhigan et al. (2017) suggested that an approach to take when trying to reconstruct diets from fatty acid signatures, in the absence of feeding experiments to derive calibration coefficients, is to treat the calibration coefficients as outputs rather than inputs. They can then be derived alongside the proportional diet representations. An issue with this approach, however, is that it is possible by judicious selection of calibration coefficients to make any selected combination of dietary inputs fit perfectly to the observed body fat distribution. Nevertheless, we utilized this approach in a slightly different way than suggested. We first set all the calibration coefficients to 1 and ran the optimisation program for 3 randomly selected index animals (1 per site). We then took the best-fit distributions and calculated the calibration coefficients that would be necessary to make the fit between the predicted diet combination and the observed body fat perfect. We then averaged these calibration coefficients across the three index animals and re-ran the optimisation program incorporating these average calibration coefficients for each fatty acid into the program.

## Results

### Fatty acid composition of the different diet sources

**Fig. 1:**
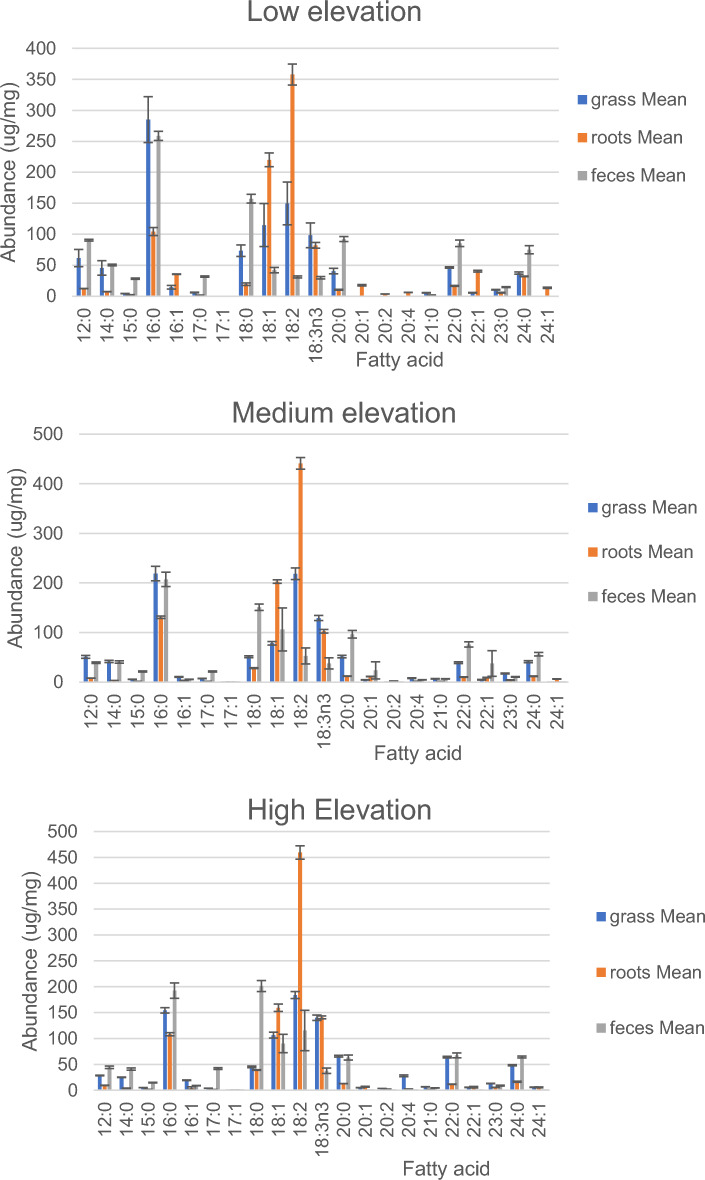
Fatty acid compositions of three different food types consumed by Plateau Pika on the Qinghai-Tibetan plateau from three different elevations (low, medium and high). Data show the ug/mg for each fatty acid species illustrating the clear differences between the foods for some of the fatty acids, and the broad comparability across elevations

We analyzed the fatty acid composition of the extracted fat in three potential food sources of plateau pika, including grass, roots and yak feces (Table 1). Coefficients of variation were generally in the range of 2 to 5% suggesting that the food sources were largely consistent within a given site. The fatty acid composition showed variable trends with elevation (Table 1). Some like lauric acid (12:0) and palmitic acid (16:0) in grass and feces, and mystric acid (14:0) in all three food types declined with elevation. While others, such as linoleic acid (18:2) and linolenic acid (18:3), increased with elevation in all three food sources. Yet others, such as stearic acid (18:0) and arachidic acid (20:0), did not have consistent trends across the different food sources with elevation. The general pattern for the three foods, averaged across elevations is illustrated in Fig. 1. Overall, the feces and grass seemed to have high levels of fatty acids with chain lengths less than 18. Only feces contained tridecylic acid (13:0), and they also had much higher levels of pentadecanoic (15:0), margaric (17:0) and stearic (18:0) acids than either grass or roots. Roots on the other hand were rich in oleic (18:1), linoleic (18:2) and linolenic (18:3) acids. Some of the longer chain fatty acids were also quite common in roots including arachidic (20:0) and behenic acid (22:0).

**Table 1:** Representation of different fatty acids in three potential food sources of plateau pika (Grass, roots and Yak feces), collected from three sites on the Qinghai-Tibetan plateau, at low medium and high elevation. Data show mean and sd of 8 replicate measures for each diet at each site

	GRASS			ROOTs			FECES		
Elev	Low	Medium	High	Low	Medium	High	Low	Medium	High
	Mean ± sd	Mean ± sd	Mean ± sd	Mean ± sd	Mean ± sd	Mean ± sd	Mean ± sd	Mean ± sd	Mean ± sd
12:00	61.7 ± 13.9	51 ± 2.9	28.6 ± 0.6	12.5 ± 0.2	8.1 ± 0.2	9.4 ± 0.3	90.5 ± 1.4	39.2 ± 1.6	44.3 ± 2.5
13:00	0 ± 0	0 ± 0	0 ± 0	0 ± 0	0 ± 0	0 ± 0	12.4 ± 0.3	4.7 ± 0.1	3.9 ± 0.3
14:00	45.7 ± 11.7	41.7 ± 2.2	24.9 ± 0.5	7.4 ± 0.2	3.4 ± 0.1	3.3 ± 0.8	50.4 ± 1.4	40.6 ± 2.4	40.9 ± 2.1
14:01	0 ± 0	0 ± 0	0 ± 0	0 ± 0	0 ± 0	0 ± 0	0 ± 0	0 ± 0	0 ± 0
15:00	4.4 ± 0.2	5.7 ± 0.3	5 ± 0.1	2.5 ± 0.1	1.5 ± 0.1	1.8 ± 0.1	28.4 ± 1	21.5 ± 1.2	14.7 ± 0.6
16:00	285 ± 37	218.9 ± 14.6	154.4 ± 5.2	104.4 ± 6.4	130.8 ± 2.3	108.2 ± 3.1	258.7 ± 7.4	207.2 ± 14.6	192.6 ± 14.9
16:01	14.5 ± 2.9	10.4 ± 0.7	19.3 ± 0.7	35.5 ± 0.4	3.3 ± 0.3	4.2 ± 2.5	0 ± 0	5.6 ± 0.4	8.9 ± 0.4
17:00	6.1 ± 0.5	7.3 ± 0.3	3.8 ± 0.1	1.9 ± 0.1	1.7 ± 0	1.6 ± 0.1	31.7 ± 1	21.5 ± 1.2	41.8 ± 1.7
17:01	0 ± 0	0 ± 0	0 ± 0	0 ± 0	0 ± 0	0 ± 0	0 ± 0	0 ± 0	0 ± 0
18:00	73.5 ± 9.2	51.2 ± 2	45.1 ± 1.6	19.5 ± 2	28.2 ± 0.9	39.1 ± 0.6	157.4 ± 7.1	151 ± 6.4	201.4 ± 10.6
18:01	114.9 ± 34.7	78.3 ± 3.6	106.6 ± 5.7	220.2 ± 11.1	202.8 ± 3.6	159.5 ± 7.1	42.1 ± 4.3	106.3 ± 43.4	90.3 ± 17.8
18:02	149.7 ± 34.5	218.6 ± 11.8	183.9 ± 6.8	357.7 ± 16.9	441.1 ± 11.8	459.5 ± 13	31.2 ± 1.6	52.8 ± 16.1	115.4 ± 39
18:3n6	0 ± 0	0 ± 0	5.3 ± 0.3	0 ± 0	0 ± 0	0 ± 0	0 ± 0	0 ± 0	0 ± 0
18:3n3	98.3 ± 19.9	129.3 ± 5.1	140.1 ± 5.2	82.2 ± 4.6	102.6 ± 3.6	140.9 ± 2.8	29.9 ± 1.9	37.8 ± 11.2	37.6 ± 5.2
20:00	40.6 ± 4.4	51.5 ± 2.5	65.8 ± 1.7	10.5 ± 0.7	12.2 ± 0.4	12.8 ± 0.2	92.2 ± 4	96.5 ± 7.6	63.1 ± 4.8
20:01	0 ± 0	4.6 ± 0.4	5 ± 0.3	17.8 ± 1.1	11.3 ± 0.3	6.7 ± 0.9	0 ± 0	23.7 ± 17.2	0 ± 0
20:02	0 ± 0	0 ± 0	3.5 ± 0.1	3.6 ± 0.1	2.3 ± 0.2	2.2 ± 0.2	0 ± 0	0 ± 0	0 ± 0
20:3n6	0 ± 0	0 ± 0	0 ± 0	0 ± 0	0 ± 0	0 ± 0	0 ± 0	0 ± 0	0 ± 0
20:04	0 ± 0	7.9 ± 0.7	27.8 ± 1.7	6.2 ± 0.2	1.7 ± 0.1	2.2 ± 0.3	0 ± 0	4.6 ± 0.2	0 ± 0
20:3n3	0 ± 0	3.9 ± 0.2	4 ± 0.1	0 ± 0	0 ± 0	0 ± 0	0 ± 0	0 ± 0	0 ± 0
20:05	0 ± 0	10.4 ± 1.2	33.3 ± 1.8	5 ± 0.6	5.6 ± 0.1	2.2 ± 0.2	0 ± 0	0 ± 0	0 ± 0
21:00	5.5 ± 0.4	6.7 ± 0.4	6.6 ± 0.2	2.1 ± 0.1	1.5 ± 0.1	1.7 ± 0.1	0 ± 0	6.5 ± 0.4	4.4 ± 0.3
22:00	46.4 ± 1.1	39.2 ± 1.8	64.1 ± 1.6	16.7 ± 0.7	10.2 ± 0.4	11.6 ± 0.4	85.4 ± 5.2	76 ± 5.2	67.4 ± 4.6
22:01	5.6 ± 0.5	4.8 ± 0.5	5.6 ± 0.4	40.5 ± 1.4	9.2 ± 0.4	5.9 ± 1.5	0 ± 0	37.6 ± 25.9	0 ± 0
22:02	0 ± 0	0 ± 0	0 ± 0	2.4 ± 0	0 ± 0	0 ± 0.1	0 ± 0	0 ± 0	0 ± 0
22:06	0 ± 0	0 ± 0	0 ± 0	0 ± 0	0 ± 0	0 ± 0	0 ± 0	0 ± 0	0 ± 0
23:00	10.5 ± 0.5	17.3 ± 0.9	13.1 ± 0.3	5.7 ± 0.6	4.1 ± 0.6	5.5 ± 0.2	14.7 ± 0.7	10.6 ± 0.6	9 ± 0.8
24:00:00	37.4 ± 1.9	41.2 ± 2	48.3 ± 1.1	32 ± 0.5	11.9 ± 0.7	16.4 ± 1.1	75 ± 6.6	56.3 ± 3.6	64.3 ± 1.8
24:01:00	0 ± 0	0 ± 0	5.8 ± 0.3	13.6 ± 1	6.4 ± 0.4	5.5 ± 0.8	0 ± 0	0 ± 0	0 ± 0

### Fatty acid composition of body fat

The fatty acid composition of stored fat averaged across all the sampled pika and mice was dominated by about ten different fatty acids (Table 2). In both species fatty acids with even chain lengths were more common than those with odd chain lengths. Fatty acids with chain lengths above 20 were uncommon averaging less than 5 ug/mg each. The most abundant fatty acids had chain lengths of 16 or 18. The biggest difference between the two species was that mice had much higher levels of oleic acid 18:1 (459 ug/mg in the mouse compared to 104.8 in the pika) while pika had high levels of linolenic 18:3 (n-3) (266.0 ug/mg) that was almost absent in the mouse (1 ug/mg). To compare profiles of the stored fat to the foods we chose all the fatty acids that had an average representation across both species of > 5.0 ug/mg (n = 10). These comprised 12:0, 14:0. 15:0. 16:0, 16:1, 17:0, 18:0. 18:1, 18:2 and 18:3 (n-3) fatty acids.

**Table 2:** Average levels (ug/mg) of fatty acids in body fat of plateau pika (n = 20) and domestic mice (n = 5)

FA	Pika	Mouse
12:0	6.4	4.1
13:0	1.3	0.0
14:0	46.6	28.7
14:1	2.4	2.1
15:0	11.5	1.3
16:0	249.9	233.4
16:1	55.1	119.6
17:0	9.9	0.8
17:1	3.3	2.1
18:0	34.0	35.2
18:1	104.8	459.8
18:2	200.9	97.0
18:3n3	266.6	1.0
20:0	1.3	1.3
20:1	1.2	8.0
20:2	1.0	2.0
20:3n6	0.0	0.0
20:4n6	1.7	2.0
20:3n3	0.0	0.0
20:5	1.0	0.6
21:0	0.0	0.0
22:0	0.4	0.0
22:1	0.0	0.0
22:2	0.0	0.0
22:6	0.6	0.9
23:0	0.0	0.0
24:0	0.0	0.0
24:1	0.0	0.0

### Predicting the diets at different sites with constant calibration coefficients.

**Fig. 2:**
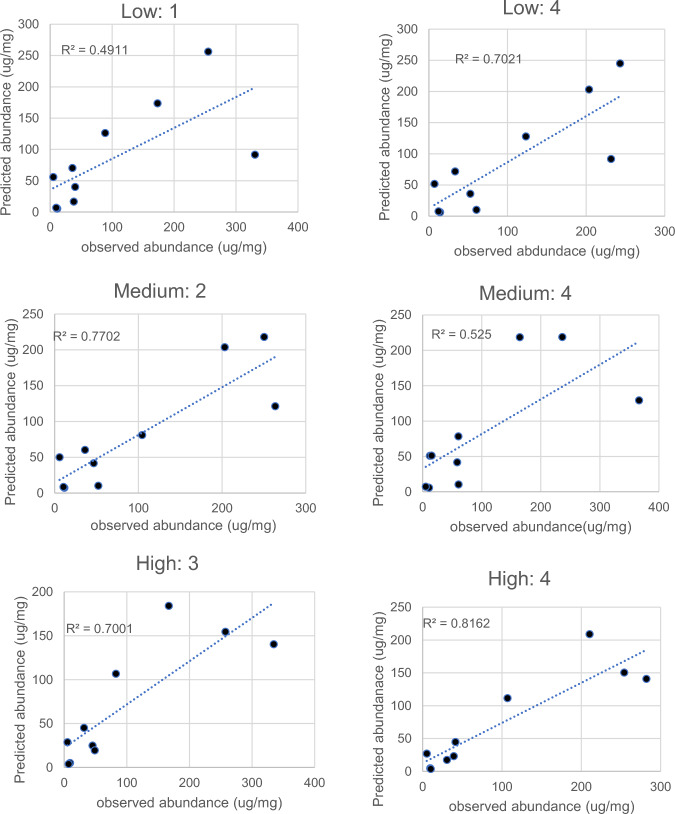
Example correlations between the prediction of the best-fit diet from the algorithm excluding any calibration coefficients and the observed fatty acid distribution in six individual pikas (2 from each elevation). Each point is a different fatty acid. Dotted lines are fitted regressions. The titles refer to the elevations and the ID numbers of the individuals (refer to Table 2). Plots for the individuals not illustrated here are in the supplementary materials Fig. 1

The predicted diet compositions for pika at each of the three elevation sites from the computer program and the summed deviations for these pika and mice fitted for each site are shown in Table 3. The squared correlation coefficients between the observed and best-fit diets are also shown. The summed deviations (ignoring signs) between the observed diets and the best fitted model are listed as best model deviations (BMD), for each individual shown alongside the best-fit diet (predicted % of Fatty acids derived from each source). The squared Pearson correlation coefficient between the best-fit model and observed distribution is also shown (r^2^). The summed deviations for the pika were substantially lower than for the mice. On average the pika best-fit summed deviations were around 350 ug/mg while for the mice the deviations were around 750. At each site the deviations were highly significantly lower in the best-fit models compared to pika (t-tests: p 3.4 × 10–7 to 7.4 × 10–8: Table 3). For each individual animal there was a strong positive correlation between the fatty acid distribution from the best predicted diet and the observed fatty acid distribution. Some example plots are shown in Fig. 2.

**Table 3:** Reconstructed diets of the pika and mice (negative control) using the best fitting algorithm, with no calibration coefficient adjustment. In addition, the predicted % of fatty acids in pika’s body fat stores derived from each food type is shown

PIKA ID	BMD	R2	Grass	Roots	Feces		MOUSE	R2	BMD
Low Elevation									
Site F 1	493.6	0.491	79	15	6		site F	0.31	757.7
2	405.8	0.525	86	11	3			0.30	759.8
3	408.4	0.509	68	16	16			0.33	785
4	309.2	0.702	65	24	11			0.28	685.1
5	353.7	0.650	83	17	0			0.42	748.9
6	302.6	0.771	82	18	0		Mean		747.3
7	216.8	0.916	67	33	0		Stdev		37.3
8	303.8	0.724	69	28	3				
Mean	349.2		74.9	20.5	4.9		t-test p =		2.44E-08
SD	85.3		8.4	7.4	5.6				
Medium Elevation									
Site H 1	328.8	0.716	92	0	8		site H	0.26	732.2
2	320.6	0.770	91	0	9			0.28	757.3
3	368.5	0.681	81	0	19			0.15	762.6
4	475.2	0.525	100	0	0			0.23	672.5
5	350.8	0.622	71	7	22			0.18	710.9
6	373.7	0.666	76	0	24		mean		727.1
7	321.4	0.770	100	0	0		Stdev		33
Mean	362.7		87.3	1	11.7				
SD	54.1		11.5	2.6	10		t-test p =		7.43E-08
High Elevation									
site X 1	310.72	0.869	86	14	0		site X	0.31	817.9
2	314.67	0.885	74	22	4			0.30	856.1
3	432.5	0.854	100	0	0			0.34	826.5
4	316.7	0.876	91	9	0			0.29	793.6
5	343.7	0.89	81	19	0			0.42	728.5
Mean	337.5		86.4	12.8	0.8		Mean		804.5
SD	53.1		9.9	8.8	1.8		Stdev		48
							t-test p =		3.32E-07

### Predicting the diets at different sites with derived calibration coefficients.

**Fig. 3:**
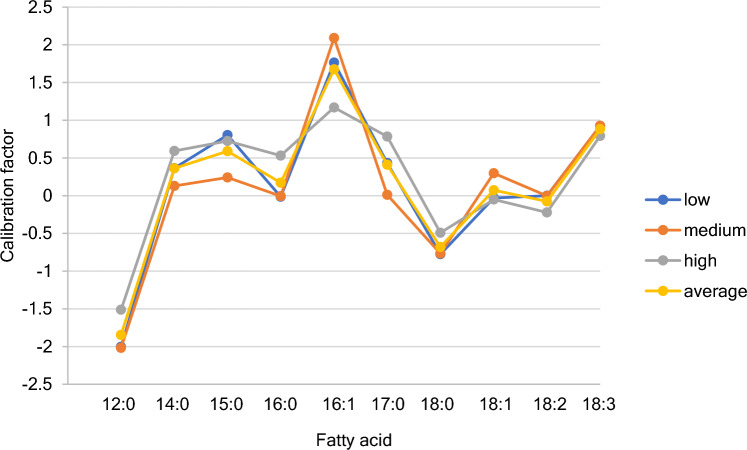
Log_e_ calibration coefficients derived for three index animals (one from each elevation). The factors relate to each of the 10 most abundant fatty acids in the diet. A positive value implies the animals discriminate against storing the given fatty acid and a negative value implies storage of that fatty acid is favored. A value of 0 implies no discrimination. The patterns were very similar across the three animals suggesting similar physiologic processes at play in the different individuals. The unlogged factors were used to derive revised predictions of the dietary sources

**Fig. 4:**
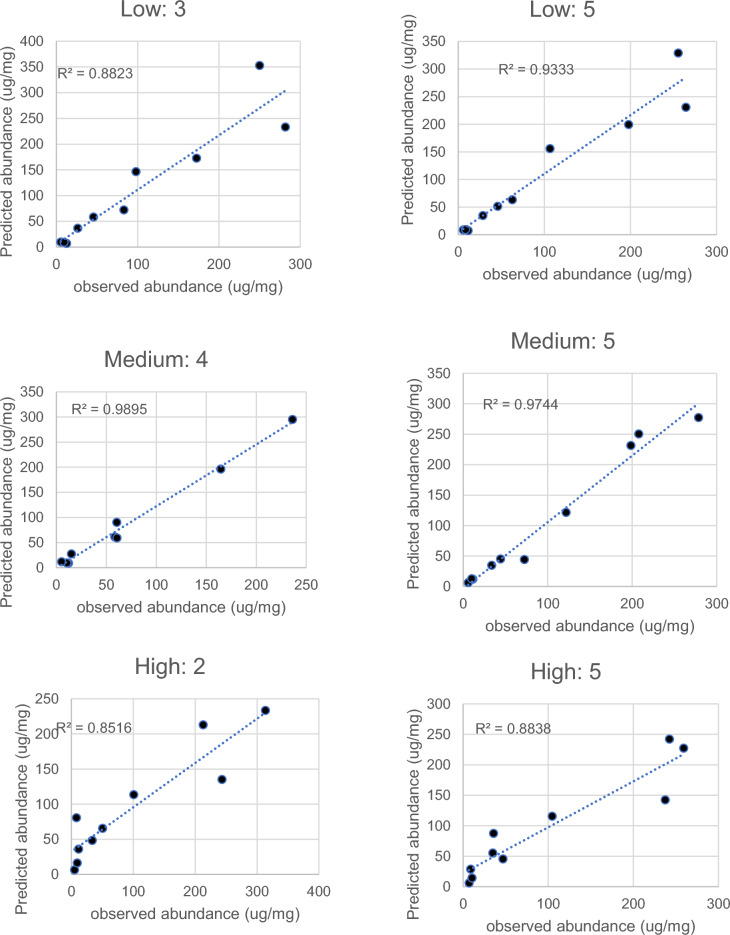
Example correlations between the prediction of the best-fit diet from the algorithm including calibration coefficients (Fig. 3) and the observed fatty acid distribution in six individual pika (2 from each elevation). The titles refer to the elevations and the ID numbers of the individuals (refer to Table 4). Plots for the individuals not illustrated here are in the supplementary materials Fig. 2

The calibration coefficients derived for the three index animals as outputs of the analysis and the log_e_ average calibration coefficients for these 3 animals across the different fatty acids are illustrated in Fig. 3. Most of the coefficients were between 0.8 and 1.7 (logged values -0.22 to + 0.53). Four were more extreme. Two were less than 0.8. For 12:0 the coefficient averaged 0.176 (logged -1.73). For 18:0 it averaged 0.537 (logged -0.621). Two were considerably greater than 1.7. For 16:1 the coefficient was 5.658 (logged + 1.73) and for 18:3 it was 2.37 (logged + 0.86). We then re-ran all the analyses using the average unlogged calibration coefficients as inputs in the analysis, rather than unknown outputs. The resultant diet assignations, best model deviations and squared Pearson correlations of predicted to observed distributions are in Table 4. Some plots showing correspondence between the predicted fatty acid levels and those observed in the body fat are illustrated in Fig. 4.

**Table 4:** Predicted diets of pika at three sites using the prediction algorithm including the derived calibration coefficients from three index animals (See Fig. 3). In addition, the predicted % of fatty acids in pika’s body fat stores derived from each food type is shown

Pika ID	BMD	R^2^	Grass	Roots	Feces
Low elevation					
Site F 1	293.9	0.794	37	45	18
2	261.4	0.847	72	18	10
3	243.65	0.882	84	15	1
4	132.4	0.959	68	28	4
5	177.59	0.933	71	26	3
6	116.27	0.969	51	33	16
7	104.5	0.976	1	54	45
8	137.5	0.955	58	37	5
Mean			**55.3**	**32**	**12.8**
SD			**26.2**	**13.2**	**14.4**
Medium elevation					
Site H 1	44.5	0.997	60	18	22
2	64.24	0.988	76	12	12
3	91.89	0.981	62	14	24
4	207.58	0.989	100	0	0
5	112	0.974	55	28	17
6	99.2	0.975	53	18	29
7	135.8	0.952	27	34	39
Mean			**61.9**	**17.7**	**20.4**
SD			**22.4**	**11**	**12.5**
High elevation					
Site X 1	191.77	0.903	76	0	24
2	336.44	0.852	51	0	49
3	172.5	0.938	99	0	1
4	186.3	0.919	79	1	20
5	236.4	0.884	63	0	37
Mean			**73.6**	**0.2**	**26.2**
SD			**18**	**0.4**	**18.1**

The pattern derived using these calibration coefficients differed from that based on the assumption of equal transfer of all fatty acids from diet to body fat (Table 3). In particular, % fat derived from yak feces increased with elevation from 12.8% at the lowest site, to 26.2% at the highest. Across all the animals at all sites the % fat derived from yak feces in the diet followed a bimodal distribution, with 6 animals deriving less than 5% and the remaining 14 deriving between 10 and 49% of their fatty acids from yak feces. The animals with very low representation were more abundant at the lowest elevation site (4/8 animals). Contrasting the pattern in the feces the fatty acids derived from roots were highest at the lowest elevation site (32%) and declined to virtually nothing (0.7%) at the highest site. Grass consumption, like the yak feces, also increased with elevation (Table 4) and was always the dominant part of the diet (55.3% at the low elevation site and 73.6% at the highest site). 

## Discussion

It was clear from the fatty acid distributions in the available diets (Table 1) and the fatty acid distributions in the body fat (Table 2), that there was not a strict 1:1 correlation between what fat the animals ingested, and what they deposited into their fat stores. In particular, there were some long-chain fatty acids that were abundant in the foods, like 22:0 and 24:0 but these were almost completely absent from the body fat. Consequently, there must be some selectivity at play between ingestion of given foods, and the deposition of fat derived from those foods into the stored body fat. This potentially compromises the ability to reconstruct diets from fatty acid signatures. Such effects could occur at different stages in the process, from selective fat uptake in the gut, through metabolic inter-conversion of fatty acid species via desaturation and elongase enzymes, either by bacteria in the alimentary tract, or by processes in the body post absorption, differential utilization and prioritization of fatty acids in triglycerides for storage and then different utilization from storage when animals are in negative energy balance. This lack of a quantitative direct correspondence between diet and stored fat has been known for at least 2 decades in marine systems (Grahl-Nielsen et al. 2003; Iverson et al. 2004). Hence, many previous studies have suggested that an accurate diet reconstruction requires derivation of calibration coefficients for each fatty acid obtained from controlled feeding studies (Rosen and Tollit 2012; Galloway and Budge 2020) that capture the differential selectivity of deposition patterns. We did not have the capacity to hold pika in captivity for long periods of time to perform such feeding trials, and it is unclear if pika could be induced in captivity to consume single diets for derivation of such calibration coefficients. The inability to perform feeding trials to derive calibration coefficients has been recognized as a foundational issue with the QFASA approach (Bromaghin et al. 2017). Even when validation experiments have been performed their validity in given environmental situations cannot be verified (Bromaghin et al. 2016).

Given our inability to derive calibration coefficients from feeding experiments we instead used two different approaches to validate the technique. First, we applied the diet fitting algorithm to an animal that we knew was not feeding on the diets in question (i.e., laboratory mice). Our rationale in doing this was to say that if the diets reconstructed for the mouse had similar total deviations across the FAs to the diet reconstructions in the pika, then we could place no faith in those predicted pika diets. In fact, the fit between pika body fat and the reconstructed diet with the lowest error was substantially better than the best fits for the mice (Table 3 and Fig. 2). This difference was largely driven by the fact the mice had virtually no linolenic acid (18:3) in their fat stores (Table 2) while this was a large feature of all the diets (Table 1) and the mice had very high levels of oleic acid (18:1) in their body fat (Table 2) while this was not particularly abundant in two of the three diet sources (Table 1).

The second approach was to utilize the suggestion of Bromaghin et al (2017) to derive the calibration coefficients as outputs rather than inputs to the algorithm. We did this for 3 animals and then used the average calibration coefficients across these three index animals to derive new predictions of the diets for all the animals (Fig. 4 and Table 4). The diets derived in this way differed significantly from the reconstructed diets assuming no differential selectivity of the different FAs (compare Table 3 to Table 4). This refined analysis revealed two broad patterns in the data. First, the intake of roots was high at low elevation, and fell to virtually zero at high elevation. This pattern was possibly because the % time that the surface ground is completely frozen increases at higher elevations, which potentially inhibits the animals from digging to access roots. Second, the % representation of yak feces and grass in the diet both increased with elevation, opposite the pattern with the roots. In general yak are only grazed at high elevation sites until the late autumn and are then brought down to the lower pastures in winter. But as an additional complicating factor the local yak herders harvest yak feces from the low pastures to dry and use as fuel for heating their houses. Nevertheless, we have some data from photo-transects suggesting that yak feces are about 10 × more abundant at low elevation sites during winter (Speakman JR, unpublished). Despite the greater density of yak dung at low elevation, the % representation in the pika diet increased as elevation increased, suggesting this was a common but not a preferred component of the diet. Nevertheless, it may still contribute to pika survival in winter.

On average, across all 3 sites, the average % fatty acids derived from eating yak feces was 18.8%. We found supportive evidence for yak feces consumption (> 10%) in 14/20 animals (70%). This was substantially higher than the 22.7% of animals where a previous study recovered yak DNA from stomach samples (Speakman et al. 2021). This difference is probably because of the different timescales that the two methods reflect. Stomach contents likely represent food intake over the previous few hours, while the deposited fat reflects intake patterns integrated over many days or weeks, dependent on the rate of body fat turnover. In mice, for example, adipocytes only turn over at a rate of about 1–5% each day (Neese et al. 2002; Rigamonti et al. 2011), so fatty acid signatures reveal fat intake integrated over several weeks. Adipocyte turnover in pika is presently unknown. In addition, DNA in yak feces may degrade after they are deposited on the surface due to the high UV light exposure at high elevations on the QTP (Speakman et al. 2021). This degradation may be significant in the period between deposition and consumption by pika, making detection of yak DNA in the pika stomachs problematic, even if they had recently consumed such material. Despite the limitations of the technique for providing a strong quantitative analysis, the current data indicate that eating yak feces may be widespread and common.

## Conclusions

Data from the QFASA method provided here combined with our previous analyses of yak DNA in pika stomach contents suggest that eating yak feces in winter is probably a common feature of pika feeding behavior, particularly at higher elevations. This utilization of feces may contribute to the fact that pikas reach their highest densities in areas where yak are grazed. Trophic relationships between these two herbivores may be more complex than hitherto envisaged. Utilizing QFASA, which has been extensively used in marine ecosystems, may be a useful tool for diet reconstruction in terrestrial ecosystems.

## Supplementary Information

Below is the link to the electronic supplementary material.

Supplementary file1 (DOCX 145 KB)

## Data Availability

The data that support the findings of this study are openly available in [Zenodo] at https://zenodo.org/records/15208895?preview=1&token=eyJhbGciOiJIUzUxMiJ9.eyJpZCI6IjYwMzk4ZGUzLTQ4MmMtNDhlNS1hZDAxLTZkYzhmMjM1MjcxNyIsImRhdGEiOnt9LCJyYW5kb20iOiJkYWMxOGQxYTUwZTFjYTMzNDliNTE1NTRjOGI1YTg3OSJ9.17Yu0v1lIpHBNIO4SH_oCEVS8cGAt8-rrIuhXnH_twWKukhsFlVtds08yCk9DfcO6c77ULTu1ckoRv0dS7hSQA

## Code availability

The Python code used in this study was in the supplementary file.
