# Supplementary material for: Quantitative fatty acid signature analysis (QFASA) to explore the diet of the plateau pika (Ochotona curzoniae) during winter on the Qinghai–Xizang (Tibetan) plateau
Source: Oecologia. 2026 Apr 9;208(4):58. doi: 10.1007/s00442-026-05893-7 (PMC13065619; doi:10.1007/s00442-026-05893-7)
Supplement: Supplementary file 1 — Supplementary file1 (DOCX 145 KB) [file 442_2026_5893_MOESM1_ESM.docx]

**Supplementary materials.**

Python code

### Program to calculate contributions of 3 diet sources to fatty acid profiles in stored fat of pika

### The fatty acid profiles of the three diets are defined in profiles x, y and z

### The program cycles through all possible combinations of these three diet sources in terms of their % contribution to the total

### A predicted profile (called p) is generated for each combination

### The observed profile (in o) is then compared to the prediction, and the absolute deviation between them calculated

### The combination of diet sources that minimises the difference between observed and predicted diets is then generated.

### The output is the combination of diet sources and the smallest deviation

### Programmed by John Speakman

### version 2.0 7th August 2024

import matplotlib.pyplot as plt

%matplotlib inline

from mpl_toolkits import mplot3d

import numpy as np

import pandas as pd

## initialise all values and arrays

x=0

devopt=5000

xopt=0

yopt=0

zopt=0

profilex = [1,1,1,1,1,1,1,1,1,1]

profiley = [1,1,1,1,1,1,1,1,1,1]

profilez = [1,1,1,1,1,1,1,1,1,1]

o= [1,1,1,1,1,1,1,1,1,1]

popt = [1,1,1,1,1,1,1,1,1,1]

diffopt = [1,1,1,1,1,1,1,1,1,1]

Lo= [1,1,1,1,1,1,1,1,1,1]

Lpopt = [1,1,1,1,1,1,1,1,1,1]

x_vals = []

y_vals = []

z_vals = []

## 0 = 12:0 1= 14:0 2 = 15:0 3 = 16:0 4 = 16:1 5 = 17:0 6 = 18:0

## 7 = 18:1 8 = 18:2 9 = 18:3

##insert profile values for grass = x, roots = y and feces = z

profilex[0] = 50.97

profilex[1] = 41.66

profilex[2] = 5.74

profilex[3] = 218.91

profilex[4] = 10.44

profilex[5] = 7.26

profilex[6] = 51.20

profilex[7] = 78.34

profilex[8] = 218.58

profilex[9] = 129.31

profiley[0] = 9.41

profiley[1] = 3.26

profiley[2] = 1.83

profiley[3] = 108.18

profiley[4] = 4.15

profiley[5] = 1.59

profiley[6] = 39.05

profiley[7] = 159.46

profiley[8] = 459.53

profiley[9] = 140.89

profilez[0] = 39.20

profilez[1] = 40.62

profilez[2] = 21.47

profilez[3] = 207.2

profilez[4] = 5.63

profilez[5] = 21.54

profilez[6] = 151.03

profilez[7] = 106.31

profilez[8] = 52.79

profilez[9] = 37.82

#insert observed profile in adipose tissue

o[0] = 3.27

o[1] = 22.21

o[2] = 1.61

o[3] = 190.71

o[4] = 127.97

o[5] = 0.73

o[6] = 17.42

o[7] = 463.12

o[8] = 152.74

o[9] = 2.11

#set up loops to cycle through all possible values of x,y and z between 0 and 100%

while x <=100:

y = 0

while y <=100:

z=0

while z <=100:

tot = x+y+z

if tot == 100:

#only proceeds to next step if total of the components = 100%

##px, py and pz are x y and z profiles multipled by their proportional representation

px = [b*x/100 for b in profilex]

py = [c*y/100 for c in profiley]

pz = [d*z/100 for d in profilez]

##generates predicted profile in p

f=0

p =[1,1,1,1,1,1,1,1,1,1]

while f <=9:

p[f] = round(px[f]+py[f]+pz[f],3)

f = f+1

## generates difference between observed and predicted profiles in diff rounded to 3dp

f = 0

diff = [1,1,1,1,1,1,1,1,1,1]

while f<=9:

diff[f]= round(o[f]-p[f],3)

f = f+1

## sum of differences across all fatty acids in dev (ignoring sign of difference)

dev = 0

f = 0

while f<=9:

dev = dev + abs(diff[f])

f = f+1

# print (x,y,z,dev)

x_vals.append(x)

y_vals.append(y)

z_vals.append(dev)

#the above line generates all the output deviations for all valid combinations of inputs

# sets devopt lower if dev lower than current optimum and updates optimal values of x,y,z

if dev <= devopt:

xopt=x

yopt=y

zopt=z

devopt= dev

popt = p

z = z+1

y = y+1

x=x+1

f=0

while f<=9:

diffopt[f]= round(o[f]-popt[f],3)

Lo[f] = np.log(o[f]+1)

Lpopt[f] = np.log(popt[f]+1)

f = f+1

print ("The lowest deviation is ", round(devopt,3))

print ("")

print ("The combination of diets to get the lowest deviation is ", "grass = ", xopt, "roots = ", yopt, "yak feces = ", zopt)

print("")

print ("The best prediction is ", popt)

print ("")

print ("The observed profile is ", o)

print("")

print ("The difference between observed and predicted is ", diffopt)

fig = plt.figure()

ax = plt.axes(projection ='3d')

ax.plot3D(x_vals, y_vals, z_vals)

plt.show()

plt.xlabel('Loge observed')

plt.ylabel('Loge best prediction')

plt.scatter([Lo],[Lpopt])

print ("Correlation matrix")

#calculate the correlation between the observed and best prediction

print(np.corrcoef([Lo],[Lpopt]))

#set up dataframe to export

data = {

"observed": [o[0],o[1], o[2],o[3],o[4],o[5],o[6],o[7],o[8], o[9]],

"best fit": [popt[0],popt[1],popt[2],popt[3],popt[4],popt[5],popt[6],popt[7],popt[8],popt[9]],

"difference": [diffopt[0],diffopt[1],diffopt[2],diffopt[3],diffopt[4],diffopt[5],diffopt[6],diffopt[7],diffopt[8],diffopt[9]],

"diet":['grass','roots','feces','','lowest dev','','','','','',],

"optimum": [xopt,yopt,zopt,'',devopt,'','','','','',],

}

dfh = pd.DataFrame(data, index = ['12:0', '14:0', '15:0', '16:0', '16:1', '17:0', '18:0', '18:1', '18:2', '18:3'] )

print(dfh)

# Exporting data as an excel file

dfh.to_excel('I:/dfh.xlsx')

**Supplementary Figure S1.** Plots of predicted against observed abundance of fatty acids for the individuals and sites not illustrated in figure 2

**Low elevation site**

**Medium elevation site**

**High elevation site**

**Supplementary Figure S2** Plots of predicted against observed abundance of fatty acids for the individuals and sites not illustrated in figure 4

**Low elevation site**

**Medium elevation site**

**High elevation site**
